# Potential barriers in healthcare access of the elderly population influenced by the economic crisis and the troika agreement: a qualitative case study in Lisbon, Portugal

**DOI:** 10.1186/s12939-017-0679-7

**Published:** 2017-10-25

**Authors:** Julia Doetsch, Eva Pilot, Paula Santana, Thomas Krafft

**Affiliations:** 1 Faculty of Health, Medicine and Life Sciences (FHML), School of Public Health and Primary Care (CAPHRI), Department of Health, Ethics and Society, Maastricht University, Maastricht, The Netherlands; 2 Centre of Studies on Geography and Spatial Planning (CEGOT), Department of Geography and Tourism, University of Coimbra, Coimbra, Portugal

**Keywords:** Health care access, Elderly, Troika, Economic crisis, Portugal, Health reform, Qualitative research, Urban health, Health inequalities

## Abstract

**Background:**

The recent economic and financial crisis in Portugal urged the Portuguese Government in April 2011 to request financial assistance from the troika austerity bail out program to get aid for its government debt. The troika agreement included health reforms and austerity measures of the National Health Service (NHS) in Portugal to save non-essential health care costs. This research aimed to identify potential barriers among the elderly population (aged 65 and above) to healthcare access influenced by the economic crisis and the troika agreement focussing on the Memorandum of Understanding on Specific Economic Policy Conditionality (MoU) in Lisbon metropolitan area, Portugal.

**Methods:**

The qualitative study is including 13 semi-structured interviews of healthcare experts, municipality authority, health care providers, negotiator of the troika agreement, hospital managers, health economists and elderly. A content analysis was performed to evaluate the interviews applying Nvivo2011 software. The barriers identified were clustered towards the five areas of the ‘Conceptual framework on health care access’ by Levesque et al. (Int J Equity Health 12:18, 2013).

**Results:**

Healthcare access for the elderly was found inadequate in four areas of the framework: availability; appropriateness; approachability; and affordability. The fifth area on acceptability was not identified since the study neither followed a gender nor ethnic specific purpose. The main identified barriers were: current financial situation and pension cuts; insufficient provision and increased user fees in primary care; inadequate design and availability of hospital care service; lack of long-term care facilities; increased out-of-pocket-payment on pharmaceuticals; limitations in exemption allowances; cuts in non-emergent health transportation; increased waiting time for elective surgery; and poor unadapted housing conditions for elderly.

**Conclusions:**

The health reforms and health budget cuts in the MoU implemented as part of the troika agreement have been associated with increasing health inequalities in access to healthcare services for the elderly population. The majority of responses disclosed an increasing deficiency across the entire National Health Service (NHS) to collaborate, integrate and communicate between the different healthcare sectors for providing adequate care to the elderly. An urgent necessity of restructuring the health care system to adapt towards the elderly population was implied.

## Background

The European economic and financial crisis has negatively impacted several European countries [1–4]. Greece, Spain and Portugal were forced to accept harsh fiscal austerity [5–8]. Despite the fact that each European country has remained diverse in their response and recovery to their country adjusted austerity measures, the effects of the economic crisis on the general population are strikingly similar [9–12]. The cut in public expenditure has most adversely affected economically vulnerable population groups [6, 13–16]. As frequently reported budget cuts in the healthcare sector, have negatively influenced health, and limited access to health care [4, 17]. Portugal is one example of how neoliberalism policy affects access to healthcare [18–21]. The Portuguese case further illustrates the far reaching consequences on public health [4, 22, 23]. The recent economic and financial crisis in Portugal left the country incapable to reimburse its government debt. To avoid insolvency the Portuguese Government was urged in April 2011 to request a €78 billion financial aid from the troika. The troika is formed by the European Central Bank (ECB), the European Commission (EC) and the International Monetary Fund (IMF) as sovereign creditors and decision group [24]. Ideological principles underlying the concept of the troika are neo-liberalism and lean government involvement, including economic liberalization policies (ie.: fiscal austerity, denationalization, and decreases of government expenditure), to enhance and stimulate the private sector’s role in the economy [25, 26]. In May 2011, a three-year Bailout Programme, the Economic Adjustment Programme for Portugal, was introduced imposing austerity measures and budget cuts in three Memoranda of Understanding between the troika and the Portuguese Government: i) Memorandum of Economic and Financial Policies (MEFP), ii) Technical Memorandum of Understanding (TMU), iii) Memorandum of Understanding on Specific Economic Policy Conditionality (MoU) [26]. General measures of the bailout programme as well as further explanation can be found in Table 1. This study focuses on the latter one, the MoU, and its consequences on health [18]. The continuous rise in public healthcare expenditure over the last decades, as a percentage of the total public expenditure (13.8% in 2011), has added to the progression of the debt in the sector and is being predicted to be the highest in the European Union (EU) by 2060 [13]. One of the objectives in the MoU was to enhance the efficiency and cost-effectiveness of the Portuguese tax funded public universal National Health Service (NHS) by introducing a comprehensive health reform aiming to achieve savings of €550 million: i) enforcing a rational use of health services and control of expenditures, ii) reducing the public spending on pharmaceuticals towards 1% of GDP by 2013 to be in line with EU average, iii) and generating further savings in hospital operating costs [27, 28]. The continuous rise in public healthcare expenditure over the last decades as a percentage of the total government budget (13.8% in 2011) has added to the progression of the debt in the sector and is being predicted to be the highest in the European Union (EU) by 2060 [13]. Healthcare reforms and austerity measures were directed towards four main areas: pharmaceuticals, primary health services, hospital services and co-payments [13] [Table 2].

**Table 1:** Background information of the troika and the bailout programme

Troika	Bailout	General objectives: Bailout
Troika’s sovereign creditors & decision group [40]: ▪ European Central Bank (ECB) ▪ European Commission (EC) ▪ International Monetary Fund (IMF) Economic Adjustment Programme for Portugal: ▪ Memorandum of Understanding on Specific Economic Policy Conditionality (MoU) ▪ Technical Memorandum of Understanding (TMU) ▪ Memorandum of Economic and Financial Policies (MEFP)	• €4.7billion cuts of public expenditure by 2014 [6] • Cuts predominantly in health care, education and social security ▪ Education: ▪ Reduction in spending by 23% from 2010 to 2012 ▪ Social security: ▪ Family allowance for families with children was reduced to 44.60€ per month (2010) • In healthcare mainly on: drug expenditure, workforce and user charges • Workforce: ▪ Further cuts of 30.000 jobs in the public sector (2013) ▪ Salary freezes (2010) ▪ Income cuts (2011–2012) ▪ Drug expenditure: ▪ Decrease from 1.55% (2010) to 1.25% (2012) and 1% (2013) of GDP ▪ Savings in public retail pharmaceutical expenditure: ▪ reductions in pricing ▪ promotion of competition ▪ electronic prescribing ▪ prescription monitoring ▪ User charges increase ▪ Primary care: from 2.25€ to €5.00€ ▪ Emergency visits for: ▪ Primary care: 3.40€ (2007) to 10.35€ (2014) ▪ Secondary care: 8.75€ to (2007) 20.65€ (2014) [11, 44, 66]	▪ Structural reforms: [44, 45] • enhance growth • generate employment • increase competitiveness ▪ A fiscal consolidation strategy • enhanced financial control over public-private-partnerships and state-owned enterprises • decreasing public debt and deficit reducing the deficit below 3% of GDP by 2014 ▪ A financial sector strategy • to protect the financial sector against deleverage

**Table 2:** Key areas of MoU’s health care reforms and austerity measures in the National Health Service (NHS), Portugal

Pharmaceuticals	Reduction in public spending
	▪ Revision of pricing system, price reduction in expenditure for Pharmaceuticals
	▪ Reduction in the regulated price increase rates for pharmacies
	▪ Reinforcement in compulsory prescription (INN prescription) of generic medicine
	▪ Formation of intensive monitoring mechanisms with evaluation and response to physicians and pharmacies
	▪ Introduction of clinical guidelines
	▪ Compulsory electronic-prescriptions (e-prescriptions) by active substances for consistent monitoring, evaluation and reporting
Primary care services	Reinforcement of provision and efficiency of the Primary care services
	▪ Equal allocation of general practitioners (GPs) throughout the country ▪ Restructuring of Health care units (ACES) into family health units ‘Unidades de Saúde Familiares’ (USFs)
	▪ Wages and services associated payments
	▪ Introduction of electronic platform of medical records assessed by primary care providers and hospitals
	▪ Increase of the number of USFs to achieve an even geographic distribution of GPs
Hospital care services	Centralization and Reorganization of public hospitals: Attainment of savings in operational costs
	▪ Merger of several hospital outpatient services to primary care units
	▪ Staff reallocation, rationalization of resources and facilities ▪ Management of staff working hours: Decrease in staff overtime compensation
Co-payments	Increase in NHS co-payments – user fees, ‘taxas moderadoras’
	▪ Revision of the of the NHS cost-sharing schemes (co-payments) to reinforce Primary care usage [see Table 5]
	▪ Automatic Indexation to Inflation of co-payment taxes
General healthcare cost reduction	▪ Fundamental revision and adjustment of accompanying exemption rules for healthcare payment
	▪ Reduction in tax allowances for healthcare expenditure by two thirds (incl. Private insurance expenses)
	▪ Revision in provision and purchasing procedures to accomplish savings by centralising procurement (i.e. reduction in transaction costs)
	▪ Cuts in non-emergency transportation to healthcare facilities

**Based on:** European Commission. The economic adjustment programme for Portugal. Brussels: European Commission; 2011 [24]

Traditionally the Portuguese health system is characterized by three parallel and intersecting public and private systems: i) National Health Service (NHS), ii) health subsystems (Insurance schemes for e.g. civil servants, military), iii) and private voluntary health insurance (VHI). The NHS covers 55–60% and the health subsystems cover 20–25% of the Portuguese population. VHI covers around 20% of the population [29, 30]. The NHS is a universal tax-financed system and provides access to healthcare for the entire Portuguese population. The NHS principally provides primary care, which functions as a gate-keeper, and specialized hospital care. Other health services (e.g. dental care, diagnostic services) are mostly delivered by private providers, nonetheless with a substantial degree of public funding [31]. In 2007, before the financial crisis, 25.7% of the total expenditure was paid by users through out-of-pocket payments (OOP), which include co-payments and direct payments, according to EUROSTAT [32]. In 2011, the MoU broadened exemption allowances in order to moderate the effects of high OOP [31]. Exemption allowances permit persons to be freed from various payments e.g. “taxas moderadoras” “moderating fees” (co-payments). These co-payments aim to moderate the use of healthcare services by reinforcing primary care utilization over emergency care utilization, through charging lower co-payments for primary care utilization. These allowances are offered for low-income groups and were established on several criteria that were primarily based on financial needs especially for socially disadvantaged groups e.g.: pregnant women, children (under 12 years), elderly receiving low pensions, chronically ill patients, persons in charge of young persons with disabilities, and persons with certain medical circumstances (e.g.: chronic diseases, organ transplant patients) [30, 33]. Table 3 illustrates the relationship between monthly pensions and exemption allowances for elderly. Even though those who receive minimum pensions are free of co-payments, they still face difficulties in paying other OOP (e.g. medication, specialist care outside the NHS) [4, 17]. In 2014, OOP still accounted for 26.8% of total health expenditure in Portugal being comparatively high in relation to the EU average of 21.8% [34].

**Table 3:** Monthly pension and exemption allowances for elderly: Portugal, National level

Monthly Pension	Exemption allowances*
Minimum pension in Portugal €385.90	Requirements for exemption allowances met
Monthly pension of lower than €628.83	Requirements for exemption allowances met
Average calculated monthly pension €1.275	No exemption allowances on pension

**Based on:** Portugal Programme Assessment European Commission, DG ECFIN. 2014 [71]

*If other medical circumstances are prevalent (e.g.: chronic diseases, organ transplant patients) exemptions from these particular payments are allowed

**Explanation**: Table 3 shows the changes introduced for exemption allowances through the MoU in 2011. The source used is dated from 2014, but the information which was retrieved is from 2011.

The austerity policy made Portugal encounter a twofold challenge of i) accomplishing long-term financial sustainability in the health care sector, and ii) simultaneously keeping the standard of health care access by enhancing the effectiveness of the system [35, 36]. Even though the aim of the MoU was to maintain universal access to healthcare, the Portuguese Observatory on Health Systems (OPSS) has expressed concerns that the austerity measures would restrict access to health services in Portugal [37]. Legido-Quigley et al. indicated a clear deterioration of access to health care for the general population after the general measures enforced by the troika [4].

The rapid increase of the elderly population in most OECD countries facing poverty and economic hardship due to the crisis, raised awareness about this particular population group [38, 39]. The influence of the MoU on pensions and income levels contributed to the increasing trend on severe risk of poverty and material deprivation among the elderly. Despite a decrease over the last years of at-risk-of poverty rate for elderly over 65, in 2015, poverty risk was still higher with 19.4% compared to the OECD average value of 15.8% [40]. Poverty adversely affecting health and being directly correlated with inequality in healthcare access is identified to be one of the main health inequity issues in Portugal with the elderly being among the most vulnerable groups, besides other (e.g. children and unemployed) [9, 19].

In 2014, elderly represented a proportion of 20.7% from the total population and in the Lisbon metropolitan area 20.9% respectively [33]. 81.6% of the elderly, 65 and above, lived in urban areas of Portugal [26]. This demographic imbalance significantly places pressure on the workforce population and provokes prominent challenges for the Portuguese health system preparedness [41]. Pre-existing inequalities for elderly can be further identified in the increased proportion of elderly reporting unmet needs for medical examination due to financial difficulties (1% in 2008 to 3.1% in 2014) [33]. The high proportion of elderly and their co-morbidities establishes them to be among the most frequent users of the NHS, particularly in terms of hospitalizations [42–44]. In 2008, before the crisis, the elderly population were the main utilizers of the public hospitals [32, 33]. In 2014, the elderly population group in Lisbon accounted for 62.6% of all hospital admissions, compared to 62.8% nationally [45].

This research was conducted in the context of the European research project ‘Euro-Healthy’ funded by the ‘Horizon 2020’ programme and contributes to the ‘Foresight for health policy development and regulation’ [46]. Lisbon was analysed in this study, as one of the two designated urban case studies of the Euro-Healthy project.

This study aimed to detect and evaluate the impact of the MoU (troika agreement) on the potential barriers to healthcare access of the elderly population in Lisbon, Portugal.

## Methods

The research was constructed on the ‘Conceptual framework on health care access’ by Levesque et al. [47] comprising the five main dimensions: adequacy, accessibility, affordability, appropriateness, and availability— and five equivalent capabilities of population groups: ability to perceive, ability to seek, ability to reach, ability to pay, and ability to engage. This framework is built on the concept of ‘patient centred access to health care’, which is based on the 2014–2020 Strategic Planning of the European Patients Forum [48]. Leveque’s framework was chosen to enable a comprehensive conceptualisation of access to health care, since it perceives access to health care as a crossing point between users and health care resources incorporating demand and supply-side-factors. These factors are essential for assessing the influence of cuts in the healthcare sector induced by the economic crisis and the troika agreement. The cuts led inter alia to centralization and reallocation of hospitals as well as to a reinforcement of the primary care services on the supply-side in health care. These measures in turn had an effect on the demand side of the patients due to lower financial resources available to pay health care services. The framework further allows for analysing the accomplishment of access to health care taking into account the entire procedure of accessing care and profiting from the services. Consequently, access is defined as “the possibility to attain and achieve suitable health care services in conditions of perceived need for care” [47]. In addition, the framework has been previously effectively applied in multiple studies i.e. on access to chronic illness care [49], and access to primary care [50] allowing for cross national and cross sectoral comparison.

A qualitative research approach was used to evaluate the potential barriers in healthcare access induced by the troika agreement, with a focus on the consequences of the MoU for the elderly population in Lisbon, Portugal. Data was collected through 13 semi-structured interviews with a cohort of healthcare experts on ageing, health care providers (i.e.: nurses, physicians), health economists, negotiator of the MoU, municipality authority, hospital manager, and elderly. Participants were approached according to their expertise and knowledge in order to meet the eligibility criteria of the study [51]. The study sample was not intended to be representative for a wide population group but instead to be exploratory to understand the perspective of diverse stakeholders. Interviewies were recruited until the attainment of the study’s purpose (reaching saturation point) [Table 4].

**Table 4:** Informants characteristics and description of function

Informant identification number (ID)	Gender	Categories	Description of function
ID1	Male	Public Health	Physician, Public health and coordinator in DGS
ID2	Male	Health Economy	Health economist and teaching Professor
ID3	Female	Municipality authority	Municipality authority in ‘Agrupamento de Centros de Saúde (ARS)
ID4	Female	Public Health	Physician, Public health doctor at Ageing institute ‘Instituto do Envelicimento’
ID5	Male	Public health	Physician and Member of the Portuguese Medical Association ‘Ordem dos Médicos’
ID6	Male	Public health	Neuroscientist and coordinator of Ageing research group
ID7	Male	Public health	Public Health Expert, Professor of Medicine and internist
ID8	Male	Hospital care	Healthcare manager; Negotiator of the MoU
ID9	Female	Elderly	89 year old women with private health insurance
ID10	Female	Primary Health care	Medical doctor in Primary Health Care
ID11	Female	Primary Health Care	Nurse in Primary Health Care
ID12	Male	Public Health	Medical doctor, Public health specialist
ID13	Female	Eldery	63 year old women with public health insurance

An interview guide with the summary of the research’s main objectives was provided for the interviewee’s prior to the interviews. The Questionnaire comprised the areas of: i) current health access for elderly, ii) the influence of the MoU from the troika agreement and economic measures, iii) policy response, iv) ageing, v) transport, vi) and accessibility of healthcare services. In order to achieve an in-depth understanding of the potential barriers perceived by the interviewees, questions were held open and merely served the purpose of structuring the interview and to give an initial impulse. The questionnaire was adapted towards the differentinterviewee’s background and context (i.e.: health care provider, health economist, muncipality authority).

The interviews had been conducted between May and July 2016 and had been audio recorded with prior consent of the participants. The variety of the experts allowed the provision of diverse insight variability in individual statements and opinions on the healthcare access barriers. The interviews conducted in English were verbatim transcribed maintaining original connotations; the interviews conducted in Portuguese were synoptically transcribed and translated.

A content analysis [52] was performed to evaluate the interviews applying Nvivo2011 software. This allowed to identify key concepts within the interviews, which were ranked by the frequency of the respondents’ reference and sorted into minor sub-categories called codes (i.e: poly-medication, out-of-pocket-payment, financial burden). Codes were sorted into categories allowing to link and relate different codes into major categories called nodes (i.e.: pharmaceuticals). This procedure permitted organizing the data into significant clusters of identified barriers in healthcare access. Barriers identified in the interviews were then categorized into the five aforementioned areas of the applied theoretical framework by Levesque et al. [47]. Table 5 serves as a visualization of the previously introduced content analysis´ process.

**Table 5:**
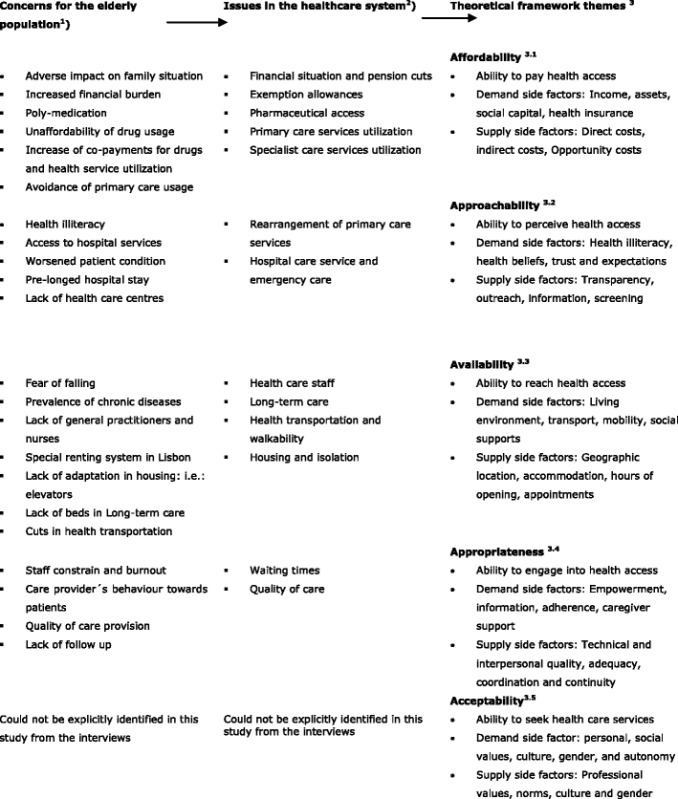
Content analysis procedure

^1^Key concepts were identified in the interviews, which were ranked by the frequency of the respondents’ reference and sorted into minor sub-categories called ´codes´

^2^The emerged ´codes´ were sorted into categories allowing to link and relate different codes into major categories called ´nodes´

^3^ **´**Nodes´ were organized into significant clusters of identified barriers in healthcare access, called ´theoretical framework themes´

^3.1^Portrays the direct and indirect costs of accessing health care services

^3.2^Discusses the attempt of health service providers to interconnect their presence and obtainable service to the population

^3.3^Refers to the opportunity of healthcare services being reachable in a timely manner

^3.4^Debates if the providing health services being timely from a curative position and appropriate in quality

^3.5^It assesses the perception of needs and desire for care of the care receiver

**Source:** Authors’own compilation

**Based on:** Levesque J-F, Harris MF, Russell G. Patient-centred access to health care: conceptualising access at the interface of health systems and populations. Int J Equity Health 2013;12:18. doi:10.1186/1475-9276-12-18

## Results

The results are arranged into four sections on: i) affordability, ii) approachability, iii) availability, and iv) appropriateness, which are based on four out of five theoretical framework themes of Levesque et al. [47]. The potential barriers to healthcare access associated with the Mou and economic crisis are summarized in Table 6. The fifth framework area, ´acceptability´*,* was not identified in this study, due to the fact that ´acceptability´ incorporates the aspects of professional values, norms, culture, and gender and assesses the perception of needs and desire for care of the care receiver. This framework area describes the ability to seek health care being influenced through personal and social values, culture, gender, and autonomy. The authors decided to exclude this area, since gender or ethnic specific purposes, as well as cultural norms or values could not be identified in the answers of the interviewees and were therefore not taken into account for this study. The informant’s identification is marked as (ID).

**Table 6:** Induced barriers in healthcare access for elderly

Access to healthcare	Category	Effect on elderly
Affordability	Current financial situation and pension cuts	○ impoverishment of the elderly population ○ dependence on family
	Exemption allowances	○ limited access for elderly with a middle income pension and especially with chronic conditions
	Pharmaceuticals access	○ restricted affordability of pharmaceuticals ○ patients with chronic diseases: poly-medication ➔ interaction of medications ➔ all required medication cannot be afforded
	Primary care service utilization	○ increase in co-payments ➔ decrease in primary care visits
	Specialist care service utilization	○ Gate keeping system: patients need to pay both fees ➔ chronically ill elderly as main users more disadvantaged
Approachability	Rearrangement of Primary care provision	○ enhanced health provision for elderly through increased efficiency ○ still major deficiencies of a sufficient provision are reported: shortage of healthcare staff ○ difficulty to access for elderly with low mobility
	Hospital care service and emergency care	○ greater efficiency in terms of diagnostic methods and quality of care provision ○ Higher pressure for healthcare staff ➔ less time for patients ○ Hospitals not patient centred but disease centred built ➔ access deficiently for elderly with co-morbidities
	Health illiteracy	○ barrier in the appropriate usage of the service for elderly ➔ lack of understanding on the usage of health care facilities and health benefits ➔ lack of engagement of elderly ➔ lack of understanding of the GP’s instructions on adequate application of pharmaceuticals
	Integration of health sectors	○ lack of follow up care ○ unnecessary stays of elderly in hospitals
Availability	Healthcare staff	○ excessive emigration ➔ less availability of health care staff ○ ➔ lack of follow-up ○ ➔ longer waiting times
	Long-term care	○ shortage in follow-up and public long-term care (despite major improvements)
	Health Transportation and walkability	○ cuts on free of charge non-emergency patient transportations ○ alternative transport: ➔ too costly ➔ too difficult for elderly with low mobility ➔ lack of adaptations (e.g. wheelchair fixture in busses)
	Housing and isolation	○ old houses mostly do not follow universal accessibility rules ○ ➔ elevators installation missing ➔ poor housing conditions: lack of heating ➔ low mobility ➔ fear of falling
Appropriateness	Waiting times	○ increased waiting times for elective surgery (e.g. hip replacement surgery)
	Quality of care	○ higher time constraints and pressure ○ ➔ impairing quality of care: less patience
	Policy response and elderly participation	○ lack of specific policy response and priority setting at the local level ○ present health care plans: ➔ still insufficient ➔ rather unspecific ➔ lacking the focus on access to health care services

### Affordability

#### Current financial situation and pension cuts

The economic crisis was indicated to have led to a great decline of economic power and impoverishment of the elderly population (ID5-ID8; ID11–13). The induced pension cuts with the MoU were identified to place in particular the elderly under a serious financial pressure.

*“In recent years economic power has declined a lot in Portugal. One of the groups which were mostly affected were the pensioners. […] The other factor is in fact the impoverishment of families and the cuts in their pensions. […]The main barriers are related to money and how the population has been losing economic power and has to have fewer children. Lots of the family’s need to support the old.”* [Translated quote] Nurse, healthcare staff (ID11)

Elderly were mentioned of being either more strongly dependent on financial support from family income to be able to afford pharmaceuticals and healthcare fees, but also increasingly elderly had to support with their pension their unemployed families after the crisis (ID6; ID8; ID9; ID11). This places elderly under a double financial burden of providing care for themselves on the one hand and on the other hand supporting their family (ID2; ID11). Elderly receiving a monthly pension above average (over €1.350) were effected by higher pension cuts (ID3;ID11) (Table 7).

**Table 7:** Pension cuts: Portugal, National level

Monthly Pension	Pension Cuts
Monthly pensions above €1.350	3.5% cuts
Monthly pensions between €3.750 and €7.546	10–15% cuts
Monthly pensions above	40% cuts

**Based on**: Portugal Programme Assessment European Commission, DG ECFIN. 2014. [71]

#### Exemption allowances

Due to the modifications and limitations of the exemption rules for health benefits several elderly lost their exemption allowance (ID4; ID8). This resulted in higher barriers to access to health care services especially for elderly with a middle income pension and chronically ill patients (ID5; ID9). Respondents negatively evaluated the exemptions from co-payments for chronically ill patients as these exemptions were limited to medications which are directly related to the chronic condition, even though chronic conditions usually require the intake of several medications due to the co-occurring diseases (multiple morbidities) (ID3;ID11).

#### Pharmaceuticals access

Informants stated that a reduction in expenses on pharmaceuticals through reinforcement of generic prescription has been achieved with the MoU hitherto (ID2;ID5). Still a significant share of pharmaceuticals was reported to be paid by the elderly patients through OOP. OOP was stated to restrict the affordability in the purchase of pharmaceuticals and to influence a fundamental problem for elderly with chronic diseases: poly-medication, the usage of four or more medications by a patient (ID2;ID5;ID9;ID10,ID13).

*“Mainly for those with chronic diseases, that have to follow daily specific medication, sometimes they even had to choose which is the most important medication, because they can’t afford to buy both, mainly diabetes, cardiovascular diseases. […] There are problems with medication, they go to this specific doctor and to the other one and all prescribe different medications and the interaction between medications is really bad.”* [Translated quote] Municipality authority (ID3)

OOP and financial constraints forced elderly to decide which drugs to purchase after the prescription of the General Practitioner (GP) (ID3;ID5). This was observed to ultimately result in a lack in quality of healthcare through ineffective treatment, severe interactions of medications, lack of monitoring and the increased risk for coronary artery diseases (ID2; ID6).

#### Primary care service utilization

The increase of the ´taxas moderadoras´(co-payments) in 2012 for the non-exempt users [Table 8] was mentioned to cause an altered healthcare utilization of the Primary health care service (ID5;ID8;ID11;ID12). The majority of respondents observed a decrease in the frequency of primary care visits by elderly and increase in the frequency of postponement of health care visits, until the utilization of the emergency care service was unavoidable (ID1;ID5;ID8;ID10-ID13). Patients at the emergency care service have been identified to appear in worse health conditions due to the pro-longed postponement in seeking health care (ID2;ID5).

**Table 8:** National co-payments in healthcare utilization for emergency and outpatient car [in Euros]

	2007	2011	2012	2013	2014
Emergency care					
Central hospital	8.75	9.60	20.00	20.60	20.65
Primary care	3.40	3.80	10.00	10.30	10.35
Outpatient care					
Central hospital	4.30	4.60	7.50	7.75	7.75
Primary care	2.10	2.25	5.00	5.00	5.00

**Based on:** Rodrigues R, Schulmann K. Impacts of the crisis on access to healthcare services: Country report on Portugal. Vienna: European Centre for Social Welfare Policy and Research. 2014; 1–51. Table 2, Co-payments for emergency and outpatient care (Euros); p.4. [9]

#### Specialist care service utilization

The prevalent gatekeeping system and increased user fees were identified to prevent elderly to seek primary care facilities in first place, since patients have to pay both fees for the GP and the specialist (ID7;ID10). Chronically ill elderly were specified to be particularly disadvantaged, since they are main user of these facilities due the high prevalence of co-morbidities (ID9;ID13).

### Approachability

#### Rearrangement of Primary care provision

The restructuring of Primary care provision through the MoU was affirmed to have enhanced health provision for elderly through increased efficiency, coordination, quality and physiological support (ID2;ID5). Health care centres *‘Agrupamentos de Centros de Saúde’ (ACES*), the basic provided community care, were rearranged into family health units *‘Unidades de Saúde Familiar’* (USFs) in order to provide service for a greater population group (ID10). Though increased provision of the USFs under the MoU was positively viewed to enable greater autonomy, efficiency, accessibility and quality in healthcare access for elderly through a more equal provision of GPs (ID2;ID11;ID12), respondents claim that the metropolitan area of Lisbon still encounters major deficiencies of a sufficient provision in USFs (ID3; ID11).

*“First, we had a package to establish health centres and in the last three years there were not made more health centres because it was very expensive for our working group. We had a program contract, signed in 2009, in which the central government would help the Câmera municipal de Lisboa to build six new health centres ‘Céntros de Saúde’. In 2016 only three Céntros de Saúde were built so far, so the planned six are in operation. In this sense we have a problem even more basic than just the effects of the crisis in access to doctors. We lack Céntros de Saúde in Lisbon.”* [Translated quote] Municipality authority (ID3)

The severe shortage of healthcare staff to work in the newly restructured USFs was indicated as a restricting health care approachability factor (ID10). Several of the restructured USFs were detected to not meet the universal accessibility rules for public buildings and therefore more difficult to access for elderly with low mobility (ID2;ID3;ID11).

#### Hospital care service and emergency care

Hospital management was centralized and rationalized with the health reforms under the MoU. This was recognized to have a potentially positive impact on health care access through a more rationale structure of the service, greater efficiency in terms of diagnostic methods and quality of care provision (ID1; ID8). However, healthcare reform and budget cuts under the MoU led to increased work pressure on the shrinking numbers of healthcare staff. (ID2; ID5;ID8).

*“[…] the lack of salaries, the pressure on working time […].We work much more now than we used to and we already work very well […]It’s big pressure on health professionals”* Public health expert (ID5)

The design of the hospitals was indicated to not be well applicable for the elderly population with chronic conditions and multi-morbidities. Hospitals are stated to be complexly built for primarily acute services and oriented towards medical specialists. Elderly, with multiple chronic diseases have to be examined in different departments of the hospital. Specialized departments are often placed far from each other and are therefore less accessible for elderly with additional potentially decreased mobility. A recent study among nurses further validated this issue of hospital services not being approperatly designed to serve elderly (ID8).

#### Health illiteracy

The high percentage of health illiteracy was frequently specified by informants to cause a great access barrier in the appropriate usage of the service in particular among the elderly population (ID1; ID5; ID7; ID8; ID11). Health illiteracy was stated to be indirectly impacted by the budget cuts under the MoU through thelack of investment on health care promotion for the elderly (ID5; ID7; ID8).

*“Health literacy is a key word […] we need people participating in this system. But to people to participate they need to know how the system is organized, need to know what this system offers local, so health literacy is a key point to elderly.”* Public Health physician (ID7)

Elderly were characterized to face barriers in access through: lack of understanding on the usage of health care facilities and health benefits, lack of engagement of elderly, and lack of understanding of the GP’s instructions on adequate application of pharmaceuticals (ID5; ID7).

#### Integration of health sectors

The deficiencies in integration and communication between primary and hospital care has according to two interviewees led a lack of follow up care, unnecessary stays of elderly in hospitals and rise in governmental health care spending (ID5; ID8).

### Availability

#### Healthcare staff

The shortage of the availability of GP’s and nurses, which has worsened under the austerity measures of the MoU in 2011, was specified as a major problem in Lisbon metropolitan area (ID2). The forced pension cuts were identified to have caused a substantial earlier retirement of about 1.500 physicians and an excessive emigration of nurses in the past five years to avoid to be affected by the step wise introduction of pension cuts under the MoU (ID5; ID9).

*“In 5 years [ehm] 1500 family physicians retired […] pension was being received…was being reduced because of the financial crisis, so if they keep working, they would receive a worst pension, then they retired early, although with a penalty, but still the pension would be worse if they carry on working[…]”* Public health expert (ID5)

The lack of healthcare staff was designated to have led to accessibility issues, lack of follow-up care and increased waiting times for the elderly (ID5; ID9;ID11;ID12).

#### Long-term care

The study sample indicated a shortage in follow-up and public long-term care (LTC) provision for elderly after hospital discharge outside acute hospitals creating a further barrier in access to services. Even though LTC continued to be partially subsidized by social security for people with lower socio-economic status, prevalence shortage of beds in public facilities resulting in long waiting lists, and a lack of staff availability (i.e.: qualified nurses) were reported as the result of reforms under the MoU (ID2;ID5).

*“There is a strong barrier in access in Portugal to long-term care, formal long-term care. This is a big issue. […] and this issues is very simple, there has been no investment in long-term care. So there is a dramatic limitation in the number of beds […] I’m talking about publicly subsidized long-term care. So you have the private sector for the people who can pay you have access. […] So for the people who cannot pay, there are huge waiting lists huge waiting times; ´cause the number of beds on the list is too low, far too low. ”* Health Economist (ID2)

LTC services were identified to be on higher policy priority agenda after the establishment of the National Network of Integrated Continuous Care ‘Rede Nacional de Cuidados Continuados Integrados’ (RNCCI) in 2006 (ID11). An appropriate provision of beds in the public sector has not been achieved yet. Even though LTC is provided in the private sector, it was signified that the majority of the elderly population cannot afford these facilities (ID2; ID4).

#### Health Transportation and walkability

The health budget cuts under the MoU were seen by respondents to alter elderly patients’ health care-seeking behaviour. Elderly were identified to attend less and avoid regular check-ups at the primary care service facilities as a result of the cuts on free of charge non-emergency patient transportations (ID10; ID11).

*“And they used to have [ehm] free [ehm] ambulances from fire man but the financials of transportations was cut because of the troika. And now they have more difficulties in going to primary care or going to hospitals.”* Public health expert (ID5)

The alternate usage of regular public transport to health care facilities was indicated to be either too costly, or too difficult for elderly with low mobility, since it requires a certain range of mobility (i.e.: when transferring). As barrier free access to public transport is still not sufficiently possible the cut in free scheduled ambulance transport created a further barrier (ID2; ID5; ID9). The difficulty of walkability in the metropolitan area of Lisbon was mentioned to limit reachability of health care facilities for the majority of elderly in Lisbon (ID4).

#### Housing and isolation

A particular housing situation is pointed out in the metropolitan area of Lisbon: old houses are rented with a special contract comprising low rent which has not been raised for decades. These houses though, mostly do not follow universal accessibility rules (i.e.: elevators installation) and reveal poor housing conditions (i.e.: lack of heating) (ID1;ID2;ID5; ID11). The introduced pension cuts by the MoU restrict elderly to change their house for alternate houses with enhanced conditions but with a higher rent.

*“The houses here in Lisbon – many are old and people are elderly and live in the same house for many years. They are small, no elevator... These people need to move to new homes that would allow them not to be isolated. We have a population that these houses pay very little income because they are already for many years in the same house […]. If they tried to leave this house, rent would be updated and the amount of [rent] would be higher […]. So these people cannot get out of these homes. […]The result is a lot of people living in isolation […]”* Healthcare staff (ID11)

Low mobility and fear of falling through missing adaptation was implied to prevent elderly to leave their home and to independently access healthcare services (ID4). Isolation of elderly was stated as a secondary financial related aspect to the economic crisis through the pension cuts.

### Appropriateness

#### Waiting times

The great increase in waiting time for specific consultations and elective surgery (i.e.: urgent cancer surgery) after the health care cuts of the MoU was determined as another main barrier in appropriateness of accessing care. After the introduction of the MoU including its cuts in the health care budget, waiting times were extended leading to an eminent access barrier to health care for the elderly (ID2; ID8).

#### Quality of care

Respondents identified that the attitude of care providers (i.e.: nurses) for the elderly as the main patient group, was influenced by the healthcare measures and reforms under the MoU (ID8). A study by Laranjeira [53] revealed that nurses perceived themselves to be less attentive to and patient with the elderly patients due to higher time constraints and increased work load deriving from the induced MoU measures, impairing the quality of care towards elderly patients.

#### Policy response and elderly participation

An overall absence of specific policy response and priority setting at the local municipality level in Lisbon on altering the barriers of elderly in health care access was observed. This absence was characterized to diminish quality of care by the majority of interviewees. Strategies such as ‘Active and Healthy ageing’ and the municipality plan for elderly ‘Plano Gerontológico municipal’ [54] as part of the ‘European innovation Partnership on active and healthy ageing’ were indicated to follow the objective of increasing participation of the elderly. However, they were all seen to be insufficient, unspecific and lacking the focus on access to health care services (ID3;ID8;ID12).

An additional verbatim demonstrates the different statements of informamts in more detail (Table 9).

**Table 9:** Additional verbatim following the structure of the results section

Theme	Quote	Informant Category
Affordability	*Pension cuts*	
	▪ “The troika agreement had a huge impact in Portugal at different levels […] clearly one of the biggest impact was on all elderly people. […] I would say it was obviously the population group that most suffered from this economic crisis at different levels […].”	▪ Public Health expert (ID6)
	▪ “I have the perception that many people have restricted access to health care or medication for economic difficulties, because there are often elderly whose pension serves to feed children and grandchildren who are unemployed, from the standpoint of care that has some impact.”	▪ [Translated quote] Primary care expert (ID10)
	*Exemption allowances*	
	▪ “Access to the National Health Service is easier for people who have very little money. […]These people have social support on health and other. Others, who do not have much more money, around 600 €, no longer get aid. This group, which in my opinion lives more in misery because they seem to have enough, but do not have money ‘cause they have to pay all the expenses themselves.”	▪ [Translated quote] Nurse, Healthcare staff (ID11)
	P*harmaceuticals*	
	▪ “On the one hand with the poly-medication for elderly, there were benefits for the consumption of generics. On the other hand elderly do not take medication properly or take medication double or the medication has interactions and after the family doctor or the nurse does not have enough time to support the elderly to use the medication properly.“	▪ Primary Care expert (ID10)
	▪ […] “people avoid to buy bills, because they don’t have money […] And you know people that are not so well informed well which is not such a good thing, but 10 pills a day, they say ‘Oh I cannot pay 10 pills I buy 5′.But then they decide by themselves …where they cut.[…] by the colours or the size or whatever “[…]	▪ Public health (ID6)
	▪ […]“many people don’t have access, to their medicines. They cannot pay. […] chronic disease who have to spend a lot on drugs, and so there is a problem of access “[…]	▪ Health Economy (ID2)
	*Primary care service utilization*	
	▪ “[…] what we noticed is that during and after the troika people go to emergency departments of the hospitals, normally they are in a worse condition, than they were before. […] people wait more time, before going for the emergency department.”	▪ Public Health expert (ID5)
	*Specialist utilization*	
	▪ “More elderly tried to contact the doctors so that they do not need to pay the moderating fee when accessing the hospital, because they are being chronically ill patients and have an inability certificate.“	▪ Primary care expert (ID 10)
Approachability	*Rearrangement of Primary care provision*	
	▪ “In primary care, we were in the course of reform and intended to be a higher quality service, with the creation of family health units, with more supply of nursing, more differentiated and more responsive to people’s needs. During the economic crisis what happened was that there were major blockages in relation to staff hires. For example, in ACES there is a very serious nursing shortage. We have been losing many doctors because the medical population is very old and is retiring and USF created were not enough. Every year we have been losing doctors, as we have fewer nurses than doctors.”	▪ [translated quote] Primary care expert (ID10)
	*Hospital care service and emergency care*	
	▪ “The hospitals are not designed to provide care of elderly people. They were […] mainly designed to […] to acute services […].The issue is now that most of our patients are elderly and most with chronic conditions. […] So in Portugal we have a low income from the elderly people, […] they are less educated than the rest of the population. […] If the population has low education they are not prepared to use our services […] we have a problem of usage and knowledge about these benefits.”	▪ Hospital manager (ID8)
	*Integration of health sectors*	
	▪ “We don’t have a real [sic] network, a really working network that provides care and so and when we talk about the integration between hospitals and Primary care, that’s a really important issue in Portugal. And actually there are lots of barriers in terms of communicating between hospital and Primary care.”	▪ Hospital manager (ID8)
Availability	*Health care staff*	
	▪ “The problem is that in many Primary care centers, there are not enough family physicians. […] This means that people have to go to the Primary care centers during the emergency hours […] so this is really a problem in terms of access, ok? In terms of waiting times […] they have no possibility to be regularly followed at the Primary care centre at the same person. The have to wait longer, they have to take the emergency hours. And this is a big issue, in Lisbon […]. This is an issue of access-- it’s not only accessing the care but access to high quality of care. Access to follow up of care […] It’s much more expensive for the system, because you are paying highly specialized people at the hospital, while you could treat the people at the Primary care centre. So it’s an incomplete inefficiency of the system. […] Primary care physicians decided to retire and to retire earlier. And they were not substituted […].”	▪ Health Economist (ID2)
	*Long-Term care*	
	▪ “I think it would be important to invest more in home support and respect on health in nursing and continuing care. I think the lack of nurses have much impact on care for the elderly.”	▪ [translated quote] Healthcare staff (ID11)
	▪ “It’s important to have the conscience that sometimes we need residential structures to elderly that can solve the problems of isolation, better life quality […]“	▪ [translated quote] Municipality authority (ID2)
	*Health Transportation and walkability*	
	▪ “In Lisbon 30% of people would have 55 year olds, would have lots of difficulties walking or going instead. […] if you have accessibility issues [ehm] this is important, really important in Lisbon. Them we have this difficult situation with mostly in the older part of the town, with the small sidewalks.”	▪ Public Health expert (ID4)
	▪ “I would say the problem of transportation was a big big issue, really. […]There was a subsidisation for the state, from the state for the transportation of urgent cases, ok? And these remained the same. […] For non-urgent cases it was restricted to the patients for who they was a clear indications of need in clinical terms and below a given amount of income, so it was a strong restriction.”	▪ Health Economist (ID2)
	*Housing and isolation*	
	▪ “[…] indirectly has to do with housing conditions then also money […] heating for instance, isolation of the housing, is really bad and we don’t have the central heating […] not having money to use heating […] Humidity and mold and things inside the house […] People with […] this kind of long contract. But that also meant, landlords didn’t do anything about the houses, They did no renovation or whatsoever […]”	▪ Public Health expert (ID4)
Appropriateness	*Waiting times*	
	▪ ***“***A part of a deficit in a hospital is the waiting list.[…] However, for an extra production there is some fixed costs that you have to put it. So enlarging waiting lists and time was one of the techniques and that had, because there is no money, you enlarge our waiting times.”	▪ Public health expert (ID1)
	*Quality of care*	
	▪ “[…] a questionnaire to the nurses, the National Questionnaire […] asking them if the care that they are proving them was friendly to elderly people. And mostly I can share with you the data they say that the services are not designed to them. And actually they are unfriendly to elderly people.”	▪ Hospital manager (ID8)

## Discussion

To the best of the author’s knowledge, this is the first study to explore and receive an in-depth understanding of various health experts’ perception on the health access barriers induced by health reforms and health budget cuts under the MoU for the elderly population in Lisbon, Portugal. This research differs from the previous research on the influence of the troika agreement, since it applies a qualitative method to study one of the most economic and social vulnerable population – the elderly aged 65 and above living in an urban setting (in our study Lisbon). The findings of this research are relevant for 81.6% of elderly, which correspond to those who live in urban areas, in Portugal [33]. The main barriers identified were: i) affordability: current financial situation and pension cuts, limitation and reduction of exemption allowances, increased OOP, limited access to pharmaceuticals ii) approachability: inadequate design and availability of hospital care service, limitations to accces caused by health illiteracy, lack of follow up care iii) availability: healthcare staff constrains, lack of long-term care facilities, cuts in non-emergency ambulance transportats, isolation, inadequate housing conditions iv) appropriateness: increased waiting times, less quality of care due to reduction of staff and increased work load, lack of adequate policy response, and elderly participation [ID1–13].

While the MoU’s fiscal austerity policy and its implementation measures have achieved budget savings for the healthcare sector, the measures have at the same time led to diminished healthcare access, as outlined in the results of this study. The sole focus on reducing government expenditure and enhancing the efficiency and cost-effectiveness of the NHS seemed to have overlooked or ignored the already fragile financial situation of a large portion of the elderly population: the individual economic consequences of the financial crisis had already led to an impoverishment of the larger parts of the crisis-ridden elderly population prior to the MoU [36]. The results of this study are in line with the findings of some earlier studies. A high utilization of preventable emergency care had been recognized in earlier (pre-crisis) evaluations of the Portuguese NHS, revealing an inadequacy of the NHS performance even prior to the crisis [4, 55]. Since then preventable hospitalization has risen by a risk of factor of 1.35 for every chronic condition [44]. Thus, the MoU attempted to reduce emergency care expenditure by reinforcing the usage of primary care through higher ´taxas moderadoras´(co-payments) for emergency care [18]. But since co-payments for primary health care services were also increased, care seeking behaviour could not be changed and thus aggravated the pressure on emergency care.After 2009 urgent in-patient stays considerably increasedas a result from unaffordable private care [44, 56]. An OCED report from 2015 reveals that 42% of in-hospital emergencies could have been treated in community or primary care seetings [43].

A supplementary study, conducted in 2013, observed that financial constraints prevented 15.1% of the population from acquiring necessary pharmaceuticals, 8.7% to attend required medical consultation, and transportation costs hindered 5.0% of the respondents to go to an essential medical examination [57]. Consequently, a noticeable worsening of self-reported access to health care due to the increases in co-payments was reported [4]. The austerity measures applied to the public health spending have been markedly harsh over a short period of time restricting access to health care services [20, 58] and led to rising health inequalities in Portugal [59]. Instruments intended to alter treatment seeking behaviour like higher user fees for emergency care failed due to the lack of corresponding instruments to support primary care instead [10, 60, 61]. Observed deficiencies in appropriateness of healthcare utilization were linked to lack of integration among health sectors, which further caused an inadequacy in follow-up care between primary and hospital care services. Further, elderly were identified to have a higher risk of potentially inappropriate intake of medication, due to the consumption of several drugs, and hence a risk of adverse drug side effects (poly-medication) [62, 63].

The reduction in health care staff both in primary and hospital services, resulting from the financial constrains under the MoU, has led to a reduced monitoring of the patients by the physicians and nurses [41]. The development of the waiting times for patients provides a mixed picture: while waiting times in general could be reduced the “maximum waiting time guaranteed” was identified to be not appropriate for several patient goups [39, 64]. For instance patients with cancer disease in urgent need for surgeries, indicated an increase in waiting times from 19.9% (2009) to 21.7% (2012) [65].

Centralization, reorganization and budget cuts of 16.6% for public hospitals, within the neoliberal merging policy of the MoU in 2011, resulted in savings in operational costs but were also responsible for causin inferior approachability of health care services [18]. The decreasing budgets of public hosptials (NHS hospitals) and reduced healthcare staff salariers triggered the emigration of hospital staff and led to a shortage in health care staff across the health system. Thus, centralization and reorganisation of hospitals, combined with low health literacy among the elderly, caused lower approachability and appropriateness in using the services [53, 56].

The restructuring of primary care services from ACES into USFs was seenpositively by respondents as it was identified to increase primary care efficiency and availability. At the same time a lack of health care centres, influenced by the shortage of physicians and nurses, was reported [27]. Overall a major deficiency in quality of care and access to continued care, as an essential sector of health care provision for the elderly, was identified as consequence of austerity measures. The application of the ‘Conceptual framework on health care access’ revealed inadequacies in health access within four out of five areas as a result of financialmeasures under the MoU. This confirms the study’s high relevance on identifying health care access barriers for the elderly. The detailed and diverse provision of information by interviewing various health care experts and elderly disclosed a mutual consensus on the insufficiency of the entire NHS system regarding elderly care. A striking lack in a comprehensive policy agenda and in strategic instruments to approach the major ageing challenges in a more direct and political way has been identified. The specified great deficiency in political priority setting of healthcare access barriers for the elderly was indicated to prevent further adjustment, regulation and modification of the NHS causing lack in quality of care and major deficiencies of the NHS.

### Recommendations

The integration and collaboration of primary and hospital care should be facilitated to avoid preventable hospital admissions. A greater reinforcement of health care centres and an increase health care staff provision would be essential to improve health for a broader population group. Therefore, available health budget must be increased and staff salary raised in order to avoid deficiencies in health care staff and its further loss to other European countries where higher salary is paid (brain drain). This measurement would enable enhanced monitoring of medication intake for the elderly due to higher staff availabilty, which is required to diminish drug interactions; hence improve quality of care. Further and greater spending on LTC, home visits of physicians, and social networks would improve access, prevent costly prolonged stays in hospitals and diminish isolation of elderly. To decrease waiting times for urgent surgeries (i.e. cancer patients), an expansion of integrated health care and greater extension of day surgery, is suggested. The lack of specific policy priority was identified to hinder adaptation and modification towards enhancement in health access for the elderly. Further effort should be placed on providing available information of the health system to tackle health illiteracy among the elderly and improve adequate usage of health care services. Moreover, greater involvement of elderly into society is identified of being a great necessity, in order to improve the identified health care access barriers.

### Limitations

The respondents might have been more susceptible towards the study’s issue since their participation has been related to their interest in the subject area. Language limitations on the interviewees and interviewer side might have been possibly predominant during the interviews and their translation. Further major limitations of the study included that the results of the study even though complemented with data and statistics are based on professional and experts reports.

## Conclusions

The implemented health reforms and health budget cuts in the MoU through the troika agreement have been indicated by the majority of respondents of being associated with increasing health inequalities in access to healthcare services for the elderly population. The identified barriers on health care access among elderly disclosed that the NHS in lacking collaboration, integration and communication between the different healthcare sectors. The great necessity of increasing the spending on health care as well as further adaptation of health services towards the elderly population was concluded.

The overall situation in Portugal is similar to other countries in Southern Europe, particularly Greece [17] and Spain [67], where the universality of health coverage, population health and existence of the welfare state has been challenged by austerity measures [10, 17, 67]. Hence, the authors would like to promote the necessity to conduct further research to the existing one in Portugal [20, 68–70] as well as other European countries experiencing the negative effects of the crisis bailout measures [1, 6, 12, 17, 67].
